# Clinico-pathological features of erythema nodosum leprosum: A case-control study at ALERT hospital, Ethiopia

**DOI:** 10.1371/journal.pntd.0006011

**Published:** 2017-10-13

**Authors:** Edessa Negera, Stephen L. Walker, Selfu Girma, Shimelis N. Doni, Degafe Tsegaye, Saba M. Lambert, Munir H. Idriss, Yohanis Tsegay, Hazel M. Dockrell, Abraham Aseffa, Diana N. Lockwood

**Affiliations:** 1 London School of Hygiene and Tropical Medicine (LSHTM), London, United Kingdom; 2 Armauer Hansen Research Institute (AHRI), Addis Ababa, Ethiopia; 3 ALERT Hospital, Addis Ababa, Ethiopia; Swiss Tropical and Public Health Institute, SWITZERLAND

## Abstract

**Background:**

Leprosy reactions are a significant cause of morbidity in leprosy population. Erythema nodosum leprosum (ENL) is an immunological complication affecting approximately 50% of patients with lepromatous leprosy (LL) and 10% of borderline lepromatous (BL) leprosy. ENL is associated with clinical features such as skin lesions, neuritis, arthritis, dactylitis, eye inflammation, osteitis, orchitis, lymphadenitis and nephritis. ENL is treated mainly with corticosteroids and corticosteroids are often required for extended periods of time which may lead to serious adverse effects. High mortality rate and increased morbidity associated with corticosteroid treatment of ENL has been reported. For improved and evidence-based treatment of ENL, documenting the systems affected by ENL is important. We report here the clinical features of ENL in a cohort of patients with acute ENL who were recruited for a clinico-pathological study before and after prednisolone treatment.

**Materials and methods:**

A case–control study was performed at ALERT hospital, Ethiopia. Forty-six LL patients with ENL and 31 non-reactional LL matched controls were enrolled to the study and followed for 28 weeks. Clinical features were systematically documented at three visits (before, during and after predinsolone treatment of ENL cases) using a specifically designed form. Skin biopsy samples were obtained from each patient before and after treatment and used for histopathological investigations to supplement the clinical data.

**Results:**

Pain was the most common symptom reported (98%) by patients with ENL. Eighty percent of them had reported skin pain and more than 70% had nerve and joint pain at enrolment. About 40% of the patients developed chronic ENL. Most individuals 95.7% had nodular skin lesions. Over half of patients with ENL had old nerve function impairment (NFI) while 13% had new NFI at enrolment. Facial and limb oedema were present in 60% patients. Regarding pathological findings before treatment, dermal neutrophilic infiltration was noted in 58.8% of patients with ENL compared to 14.3% in LL controls. Only 14.7% patients with ENL had evidence of vasculitis at enrolment.

**Conclusion:**

In our study, painful nodular skin lesions were present in all ENL patients. Only 58% patients had dermal polymorphonuclear cell infiltration showing that not all clinically confirmed ENL cases have neutrophilic infiltration in lesions. Very few patients had histological evidence of vasculitis. Many patients developed chronic ENL and these patients require inpatient corticosteroid treatment for extended periods which challenges the health service facility in resource poor settings, as well as the patient’s quality of life.

## Author summary

Leprosy reactions (Type 1 and 2) are important causes of nerve damage and illness. Erythema Nodosum Leprosum (ENL) also called type 2 reactions is a severe multisystem immune-mediated complication of borderline and lepromatous leprosy. ENL causes high morbidity and mortality and usually requires urgent medical attention. ENL can occur before, during, or after completion of MDT. The diagnosis and treatment of ENL is largely based on clinical symptoms. However, the clinical symptoms are heterogeneous and may vary from patient to patient. Although thalidomide is an effective drug for ENL treatment, it is not available in many leprosy endemic countries including Ethiopia. In spite of its adverse effects, in many endemic countries corticosteroid is the only available drug for ENL treatment, usually being used for prolonged periods. Therefore, alternative and effective drugs are required to reduce the burden of ENL. To establish which drugs will be effective in the treatment of ENL it is necessary to have a clear picture of the clinical and histological features of the disease. We systematically documented these features of ENL and compared them with matched non-reactional LL controls. Thus, the findings will help to develop better ENL diagnosis and treatment options.

## Introduction

Leprosy is a disease caused by *Mycobacterium leprae*, an intracellular acid-fast bacillus[1]. It mainly infects the skin and peripheral nerves[2]. The disease manifests with a spectrum of clinical pictures ranging from the localized tuberculoid leprosy (TT) to the generalized lepromatous leprosy (LL) types forming the two poles of the five point spectrum [3].

Erythema nodosum leprosum (ENL) is an immune-mediated inflammatory complication affecting about 50% of patients with lepromatous leprosy (LL) and 10% of borderline lepromatous (BL) patients [4–6]. ENL can occur before, during or after successful completion multi-drug therapy (MDT). The onset of ENL is acute, but it may pass into a chronic phase and can be recurrent [7].

ENL affects multiple organs and causes systemic illness [8].It is clinically characterized by the occurrence of crops of tender skin lesions [9]. Histologically, neutrophils are considered the hall mark of ENL[10]. The histology of ENL lesions shows an intense perivascular infiltrate of neutrophils throughout the dermis and subcutis [10]. However, not all clinically confirmed ENL cases have neutrophilic infiltration in lesions[11].

The underlying immunologic mechanisms of ENL have not been fully understood. The hypothesis of ENL as an immune-complex mediated disease proposed in the 1960s has yet to be supported by definitive evidence. Granular deposits of immunoglobulin and complements in the dermis of ENL lesion has been found by using direct immunofluorescence techniques which were absent in non-reactional LL lesions [12–14]. However, some investigators have reported the presence of immunoglobulin and complement deposits in ENL lesions as well as in LL lesions [15–17].

The contribution of cell-mediated immunity in the pathogenesis of the disease has been suggested but not supported by definitive evidence[18]. Several studies [19–23] have reported increased percentage of CD4^+^ T-cells and reduced CD8^+^ T-cells with an increased CD4^+^/CD8^+^ ratio in patients with ENL compared to patients with non-reactional lepromatous leprosy. Other studies have however, also reported a reduced CD4^+^/CD8^+^ ratio and increased percentage of CD8^+^ T-cells in patients with ENL compared to patients with LL [24].

The inflammatory condition of ENL may cause significant morbidity and mortality if it is not treated on time.[25]. In Ethiopia, patients with ENL are treated with corticosteroids for several months or years. Many patients require high doses of prednisone to control inflammation which could lead to complications. A significant proportion of deaths associated with long-term use of these drugs has been reported [25].

Having awareness of the diverse clinical features of ENL is useful for the accurate diagnosis and successful management of the disease. However, there are only few prospective studies describing the clinical features and there relative frequencies in ENL. A cross-sectional international multicentre study of the clinical features of ENL including 292 patients in 7 countries has reported that a significant number of patients had extra-cutaneous pathology such as peripheral oedema, large joint arthritis, lymphadenitis, and orchitis [9].

We set up a case control follow up study to investigate the clinico-pathological features of ENL. We compared the clinical and histological features in patients with ENL reactions to matched uncomplicated non-reactional LL patient controls before and after prednisolone treatment of ENL cases. ENL patients have diverse clinical manifestations. Therefore, prospective documentation of the clinical manifestations of patients with ENL is useful for accurate diagnosis of ENL. Unlike previous cross-sectional studies, in the present study we obtained clinical data and clinical sample (skin biopsy) from cases (ENL) and controls (LL) before, during and after treatment. The controls were matched with cases with respect to age, sex and duration of leprosy diagnosis. Hence, the present findings are more informative and show the dynamics of clinical features of ENL before and after treatment.

## Materials and methods

### Ethics statement

Informed written consent for blood and skin biopsies were obtained from patients following approval of the study by the Institutional Ethical Committee of London School of Hygiene and Tropical Medicine, UK, (#6391), AHRI/ALERT Ethics review committee, Ethiopia (P032/12) and the National Research Ethics Review Committee, Ethiopia (#310/450/06).

### Study design

A case control study was conducted between December, 2013 and October, 2015 at All Africa Leprosy and, Tuberculosis Rehabilitation and Training Centre (ALERT) Hospital, Ethiopia. This is the main leprosy specialized hospital in Ethiopia. Hence, it is an ideal hospital to obtain referred leprosy patients from all regions in the country.

### Patient recruitment and data collection

Children below 18 years old, adults above 65 years old, pregnant and lactating mothers, patients with other clinical forms of leprosy (TT, BT, BB, BL and T1R) were excluded from the study. Forty-six untreated patients with ENL and 31 LL controls were enrolled into the study and followed for 28 weeks. The controls were age and sex matched with cases (ENL).

ENL was clinically diagnosed when a patient with LL leprosy had painful crops of tender cutaneous erythematous skin lesions [5]. Lepromatous leprosy was clinically diagnosed when a patient had widely disseminated nodular lesions with ill-defined borders and BI above 2 [7].

New ENL was defined as the occurrence of ENL for the first time in a patient with LL. The nature of ENL was defined as acute for a single episode lasting less than 24 weeks while on corticosteroids treatment, recurrent if a patient experienced a second or subsequent episode of ENL occurring 28 days or more after stopping treatment for ENL and chronic if occurring for 24 weeks or more during which a patient required ENL treatment either continuously or where any treatment free period had been 27 days or less [7].

Clinical data were collected using a standard form that had been developed by the Erythema Nodosum Leprosum International STudy (ENLIST) group. Demographic, clinical and laboratory data were recorded including evidence of any nerve function impairment (NFI) using voluntary muscle and Semmes-Weinstein monofilament sensory testing. Nerve function impairment (NFI) was defined as clinically detectable impairment of sensory or motor nerve function. New NFI was defined as NFI present for less than six months[26]. The bacterial Index (BI) at leprosy diagnosis was obtained for all recruited patients. BI at ENL reaction was also obtained at enrolment.

Six millimetre skin biopsies were obtained from each ENL case before and on 24^th^ week after prednisolone treatment of ENL cases. Similarly, 6mm biopsy was obtained during enrolment and on the 24^th^ week of recruitment from matched non-reactional LL controls. Biopsies were taken from the active erythematous new skin lesions in all patients with ENL and from nodular LL lesions. Biopsies were obtained from the same area for cases and control. Biopsies were stored in 10% formalin until processed. Sections were stained with Haematoxylin and Eosin stain and examined by two histopathologists independently. The pathologists were not aware of the clinical diagnosis. Bacterial index (BI) was obtained for each patient as a routine investigation.

When a polymorphonuclear neutrophilic infiltrate on the background of a macrophage granuloma accompanied by oedema and often with evidence of vasculitis and/or panniculitis was seen, the sample was classified as ENL. The presence of macrophage and foam cell collections with numerous bacilli interspersed with sparse number of lymphocytes in histological sections was defined as LL [27].

### Statistical analysis

The anonymised clinical and Histopathology data were entered into an Excel database and analysed using Stata 14 version 2 and SPSS 23 version 1 Statistical Software. Depending on the nature of the variable and the normality of the data, either parametric or non-parametric analysis was used. Categorical variables were analysed by non-parametric methods and normally distributed numerical variables with parametric methods. Whenever mean is used for comparison, data presentation has followed the form of mean ± standard error of the mean (SE). The level for statistical significance was set at α = 5% with 95% confidence interval.

## Results

### Demographic and clinical characteristic of study subjects

Clinical data were obtained on 77 patients (46 LL patients with ENL reactions and 31 non-reactional LL patients) at recruitment (Table 1). The male to female ratio was 2:1 with a median age of 27.5 [range: 18–56] years in patients with ENL and nearly 3:1 with a median age of 25.0 [range: 18–60] years in patients with non-reactional LL controls. The age range of females in both groups was relatively narrow (18–35 years) compared to males (18–60). More than half of the patients with ENL had previously been treated with MDT. Half of the patients with ENL had acute ENL at the time of enrolment with mean BI 3.9 ±0.205 SE (standard error). Recurrent ENL cases had the highest mean BI (4.9 ±0.409 SE) at leprosy diagnosis whereas acute and chronic cases had comparable mean bacterial index (BI) (Table 1).

**Table 1:** Demographic and clinical characteristics of study subjects at enrolment.

Variables		ENL (n = 46) n (%)	LL (n = 31) n (%)
Sex	Male	31 (67.4)	23 (74.2)
	Female	15 (32.6)	8 (25.8)
Median age in years (range) group		27.5 (18–56)	25.0 (18–60)
Median age in years (range) Male		28 (18–56)	26.0 (18–60)
Median age in years (range) Female		26.7 (18–35)	21.0 (18–30)
MDT status	No previous MDT	10 (21.7)	22 (71.0)
	Current	9 (19.6)	8 (25.8)
	Completed	27 (58.7)	1 (3.2)
HIV status	Positive	0 (0.0)	0 (0.0)
	Negative	46 (100.0)	31 (100.0)
Duration of current ENL symptom (Episode) Mean ± SE [days]		6.8 ±0.491 (range: 1–15)	-
Clinical status at recruitment			
ENL type	Acute	23 (50.0)	-
	Recurrent	5 (10.9)	-
	Chronic	18 (39.1)	-
LL type	New	-	23 (74.2)
	Relapse	-	5 (16.1)
	Defaulter	-	3 (9.7)
BI at diagnosis, Mean ± SE (range)			
ENL	Acute	3.9 ±0.205 (2–6)	-
	Recurrent	4.9 ±0.409 (4–6)	-
	Chronic	3.7 ±0.103(3–4)	-
LL	Untreated (new)		4.1 ±0.259 (2–6)
	Relapse		4.2 ±0.330 (4–5)
	Defaulter		4.9 ±0.150 (4–5)

**Fig 1:**
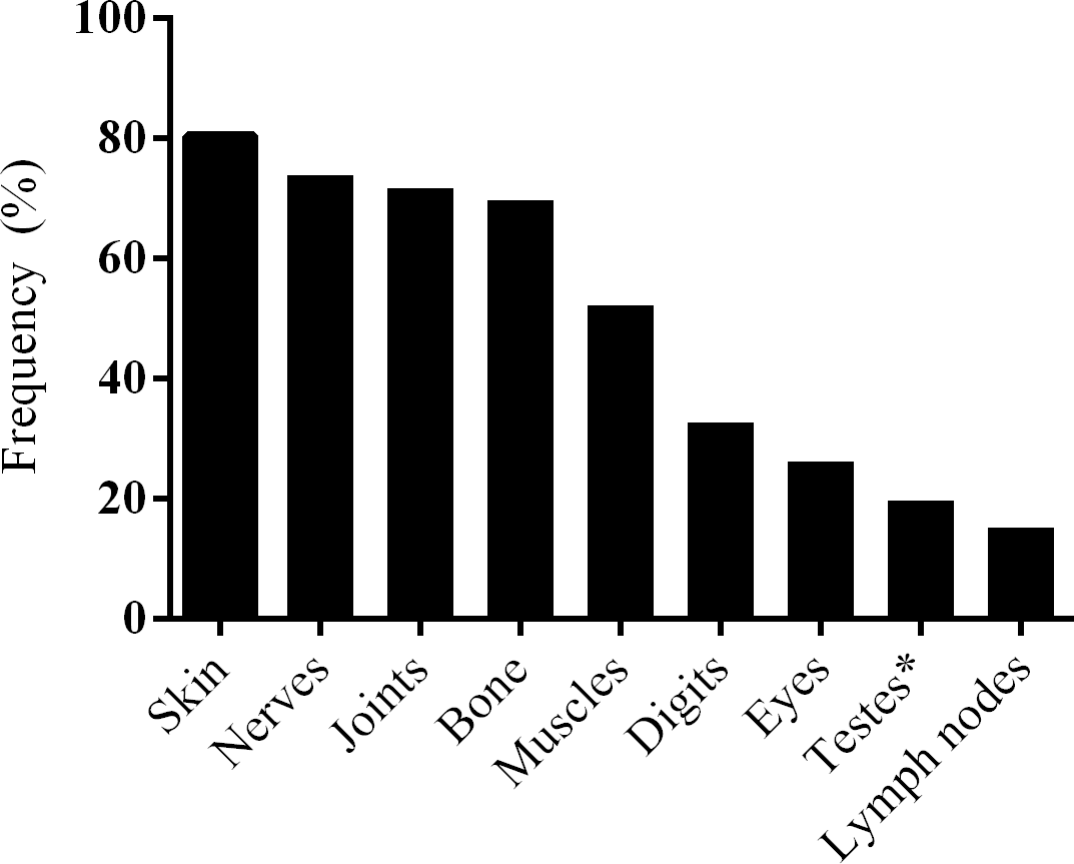
Location of pain in the patients with ENL. *value is among 31 males.

Pain was the most common symptom reported by patients with ENL. Ninety-eight percent of the patients with ENL had pain at enrolment. About 80% of the patients with ENL had reported skin pain and more than 70% had nerve and joint pain during enrolment. Other pain sites reported include bone, digits, eyes, muscles, lymph nodes and testes (Fig 1).

**Fig 2:**
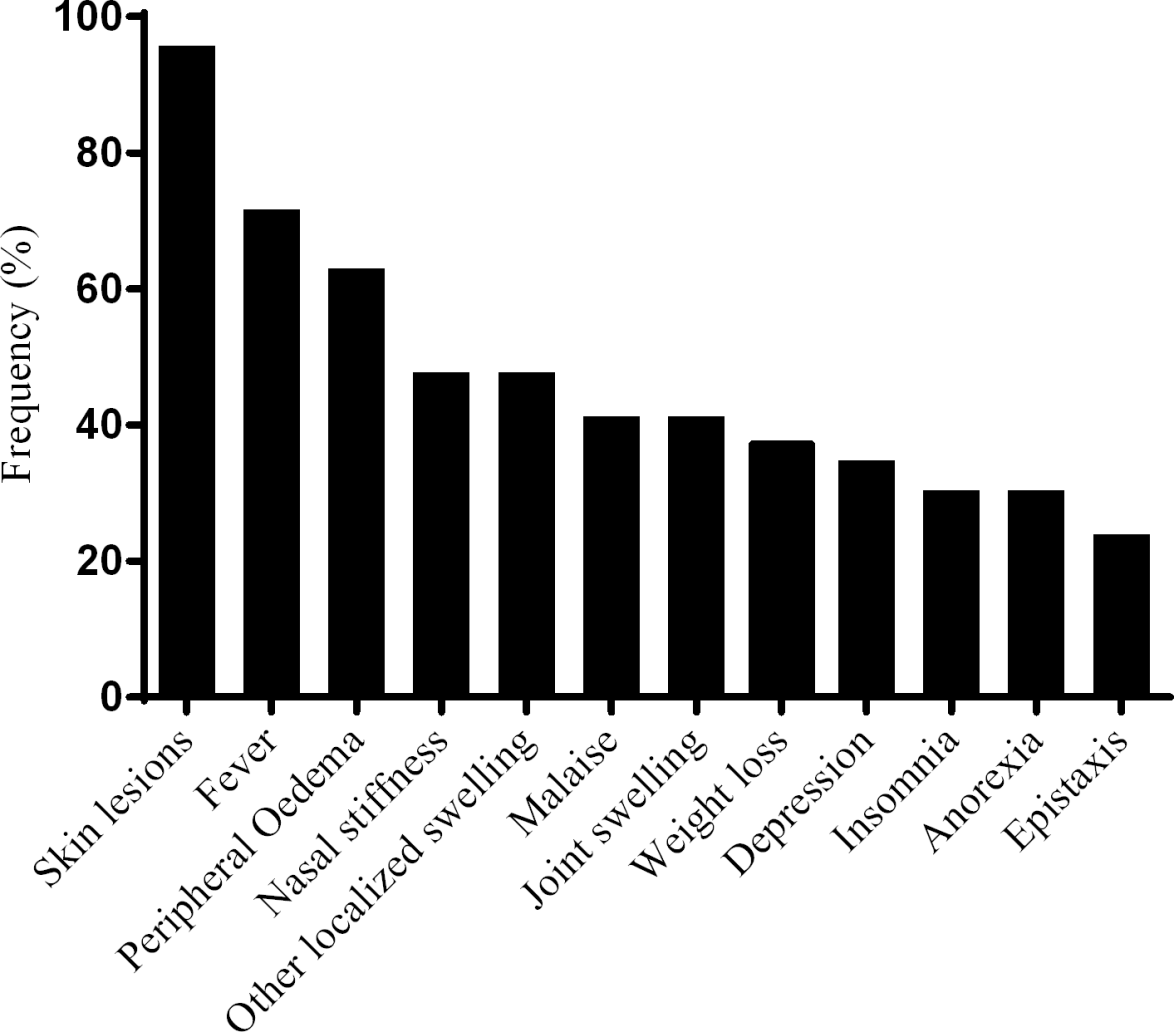
Symptoms other than pain in patients with ENL.

Fever was reported by 31 (71.7%) patients with ENL. Sixteen (34.8%) patients with ENL reported depression and 47.8% nasal stuffiness. Other reported symptoms included peripheral oedema, insomnia, anorexia, weight loss, joint swelling and malaise (Fig 2).

**Fig 3:**
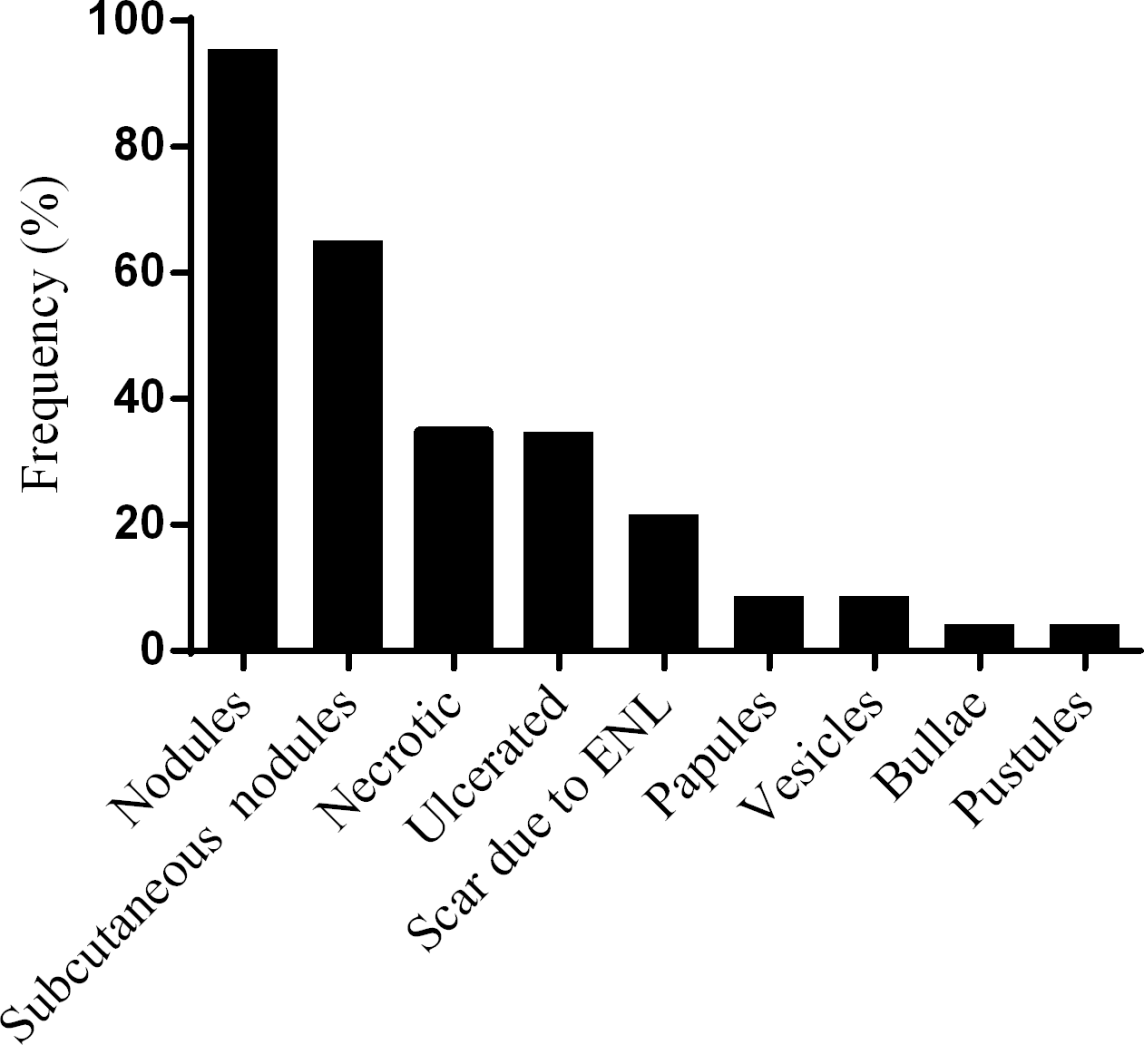
Frequency of the different skin lesions in patients with ENL.

About 96% individuals had nodular cutaneous lesions, about two-third had subcutaneous nodules and a quarter of patients had scar. While one-third of the patients had ulcerated lesions, only 4% had necrotic lesions. Eight patients (17.3%) had vesicles, bullae or pustular lesions (Fig 3).

In most patients with ENL (73.9%), the number of skin lesions recorded at the time of enrolment was between 11 to 50. Few patients had five or less skin lesions. Almost all patients (97.8%) had skin lesions on the upper limbs. Many patients also had skin lesions on the lower limbs (95.7%) or on the head and neck (63.0%). Half of the patients reported reduced nerve sensation. Paraesthesia and hyperaesthesia were reported by 13% and 23.9% of patients respectively (Table 2).

**Table 2:** Other clinical pictures in patients with ENL at enrolment.

Number of skin lesions		number	%
	<5	3	6.5
	6–10	4	8.7
	11–20	18	39.1
	21–50	16	34.8
	>50	5	10.9
Location of skin lesions	Head/neck	29	63.0
	Trunk	18	39.1
	Upper limbs	45	97.8
	Lower limbs	44	95.7
Nerve symptoms	Reduced Sensation	23	50.0
	Paraesthesia	6	13.0
	Hyperaesthesia	11	23.9
	Weakness	35	76.1
Nerve function impairment (NFI)	Old	24	52.2
	New	6	13.0
Organs involved in ENL			
Oedema	Hand	26	56.5
	Face	16	34.8
	Lower limbs	22	47.8
Dactylitis		1	2.2
Large joint Arthritis		7	15.2
Small joint arthritis		13	28.3
Conjunctivitis		2	4.3
Lagophthalmos		1	2.2
Scleritis		4	8.7
Lymph node		7	15.2

More than half (52.2%) of patients with ENL had old nerve function impairment (NFI) while 13% had new NFI at the time of enrolment. Facial oedema was reported in 56.5% of the patients with ENL and nearly half (47.8%) of the patients had oedema on their lower limbs. Other organs involved in the patients with ENL were small joint arthritis (28.3%), large joint arthritis (15.2%), conjunctivitis (4.3%), lagophthalmos (2.2%), scleritis (8.7%), lymph node (15.2%) and dactylitis (2.2%) (Table 2).

### Histopathological features of study subjects

**Fig 4:**
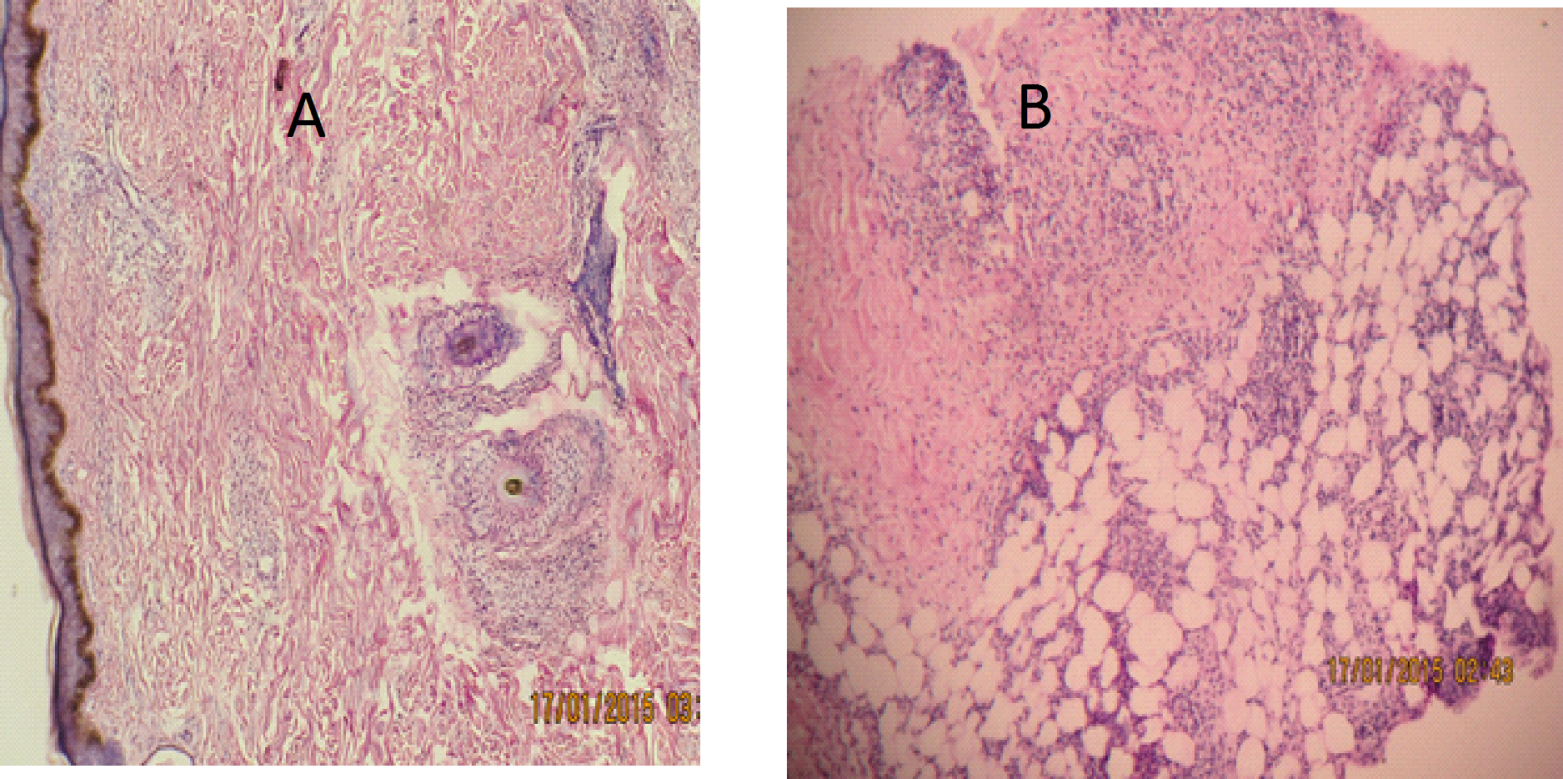
H &E stained skin biopsies: A. Histopathology of LL lesion without reaction: reticular dermal infiltration of lymphocytes, flat epidermis and foamy histiocytes.B. Histopathology of EN lesion: flat granular PMN infiltration with perivascular lymphocytic infiltration and lobar panniculitis. H & E staining x40.

Paraffin- embedded sections of skin biopsy samples from ENL and LL lesions were examined by a histopathologist (Fig 4). Neutrophils infiltration was noted more ENL lesions (58.9%) than LL lesions (14.3%) before treatment (P = 0.004). Lymphocytes infiltration was recorded in all ENL and LL lesions. Foamy histiocytes were more frequently seen in LL lesions (95.3%) than in ENL lesions (85.3%) although the difference was not statistically significant at enrolment. After 24 weeks treatment of ENL, the percentage of foamy histiocytes was significantly decreased in ENL cases (42.2%) compared to LL cases (85.7%) (p = 0.001). Panniculitis was diagnosed in 62.5% of lesions from patients with ENL reactions. After 24 weeks of ENL treatment, neutrophils infiltration was noted in 5 biopsies from patients with ENL reactions, lymphocytes infiltration was seen in 20 biopsies of patients with ENL (Table 3).

**Table 3:** Histopathological features of study subjects before and after treatment.

Diagnosis	Before treatment n (%)			After treatment n (%)		
	ENL n = 34)	LL (n = 21)	P-value	ENL (n = 33)	LL (n = 21)	P-value
Neutrophil infiltration	20 (58.8)	3 (14.3)	0.004*	5 (15.2)	7 (33.3)	0.058
Lymphocytes	34 (100.0)	21 (100.0)	0.984	20 (60.6)	21 (100.0)	0.006*
Foamy histiocytes	29 (85.3)	20 (95.3)	0.627	14 (42.2)	18 (85.7%)	0.001*
Eosinophils and mast cells	6 (17.6)	5 (23.8)	0.284	1 (3.0)	4 (19.0)	0.06
Vasculitis	5 (14.7)	4 (19.0)	0.079	2 (6.1)	2 (9.5)	0.841
Necrosis	2 (5.9)	2 (9.5)	0.068	2 (6.1)	10 (47.6)	0.004*
Panniculitis	10 (62.5)δ	0 (0)	<0.001*	6(75.0%)ε	0 (0)	0.001*

δ; n = 16. ε; n = 8.

* statistical test significant at *P = 0*.*05*.

## Discussion

The number of male patients with ENL recruited to the study was twice the number of female patients and similar to a five-year retrospective data (2008–2013) which showed the number of male to female ratio to be 1.7:1 [7]. In our study, the median age for male and female patients with ENL was 28.0 and 26.7 years respectively. Both male and female patients with ENL were relatively older than the LL patient controls (median age: male = 26 years, female = 21 years). The slight difference in median age between the two groups could be explained by natural the course of the disease. Patients usually develop ENL reaction after having either LL or BL clinical forms for some time. Interestingly, the age range of females in both groups was relatively narrow (18–35 years) compared to males (18–60 years) indicating that either younger females are more likely to have access to health institutions for various reasons than older females in low-income countries where health facilities are relatively inadequate[28] or ENL is relatively common among younger females of child bearing age due to various biological reasons [29–31].

Our data confirm that a significant proportion of cases had chronic ENL (39%). This implies that these patients require, in our setting, corticosteroid treatment for extended periods, often at high doses… But high doses of corticosteroids do not always control the inflammation and also pose life-threatening risks for patients [9, 32, 33]. Chronic ENL cases are a burden to referral hospitals in these resource poor settings. as well as to their communities. A study in rural India has shown that families with at least one ENL case incur loss of more than 40% of total household income compared to families without ENL case due to out of pocket expenditure for treatment-seeking (direct cost) and loss of income resulting from reduced productivity (earning potential) of household members (indirect cost). This implies that households affected by ENL face significant economic burden and are at risk of being pushed further into poverty [34].

In this study, several cutaneous manifestations of ENL were documented highlighting the heterogeneous nature of ENL clinical manifestation. Pain was was a symptom reported by 98% of the patients. Most patients had skin pain (80.4%), nerve pain (73.9%), joint pain (71.7%) and bone pain (69.2%). The most frequent site of pain due to ENL in our study was the skin which is explained by the fact that 95% of patients with ENL had skin lesions. Our finding is in agreement with a previous report [7]. Bone pain was reported in two-third of our study patients which is higher than the previous report [7]. The difference between the two studies is likely due to the retrospective nature of the previous study which was not reliant on case note recording unlike the current study.

The nerve function impairment (NFI) was reported in 65% of our study patients, which was higher than the 51.3% NFI in six countries as reported by Walker et al [35]. Among the 65% of patients reporting NFI, 80% of them had old NFI. This highlights the prevalence of NFI in patients with ENL the high risk of developing permanent disability. A study by Santos Santos, de Mendonça Neto [36], in northern Brazil had identified NFI and leprosy reactions as the main risk factors associated with the development of disability in leprosy patients. The same authors reported that NFI was strongly associated with physical disability in children under 15 [37]. In our study, 50% of patients with ENL had WHO disability grade-1 (G1D) while 4.3% had Grade- 2 disability (G2D). The proportion of grade 2 disability was lower than the national figure (10.2%) in 2014 [38].

Histopathologically, neutrophil infiltration was noted in 58.8% of patients with ENL compared to 14.3% in LL controls before treatment. This confirms that a neutrophilic infiltration cannot be used as the sole histological marker for ENL The absence of neutrophil infiltration has been reported in 36% of ENL skin lesions in Pakistani patients who had classical signs and symptoms of ENL[11]. Similarly, a cross-sectional study on the histological features of leprosy reactions in Indian patients by Sarita, Muhammed [39] showed that 43% ENL skin lesions did not have histological evidence of neutrophil infiltration. Our findings agree with these two studies. Previous studies by others [40–42], reported finding neutrophil infiltration in all ENL lesions. The varying reports of neutrophil infiltration in ENL lesions could be attributed to several factors. If the definition of ENL includes the presence of neutrophils in the case definition then all cases will have it, as did Aldhe *et al* who investigated the presence of cellular neutrophil infiltration on histologically confirmed ENL cases [42]. Delay between the onset of reaction and the timing of obtaining the biopsy in those without neutrophilic infiltrate, as dermal oedema may be missed in older reactional lesions could cause these differences. Discordance between pathologists and standard operating procedures (SOPs) of slide preparations are also potential areas that should be further investigated to evaluate their impact on the findings of neutrophil infiltration in tissue sections. Previous reports suggested vasculitis as part of ENL reaction commonly seen in Indian patients [43], only 5(14.7%) of our patients had evidence of vasculitis. Similar observations had been made by Sarita et al and Adhe et al [39, 44].

Inclusion of a large number of patients with ENL and LL controls was one of the strengths of this study. The other strength of the study had been that clinical data were obtained from each patient three times unlike the previous cross-sectional studies. A weakness of the study is that there may have been biased recruitment because of the need to have good follow–up of patients.

In conclusion, we have shown that skin, nerve and joint pain are the most common clinical symptoms reported in patients with ENL. These clinical conditions are usually difficult to manage with corticosteroids at referral Hospitals. Most of our patients with ENL then developed chronic ENL and these patients require in patient corticosteroid treatment for extended periods which challenges the health service facility in resource poor settings. More than half of the patients with ENL had old NFI which indicates that these patients are at a higher risk of developing permanent disability. Hence, better attention to care and NFI needed in these patients.
